# Ammonium tetrathiomolybdate in the decoppering phase treatment of Wilson's disease with neurological symptoms: A case series

**DOI:** 10.1002/brb3.1596

**Published:** 2020-03-22

**Authors:** Oriol De Fabregues, Jaume Viñas, Antoni Palasí, Manuel Quintana, Ignasi Cardona, Cristina Auger, Víctor Vargas

**Affiliations:** ^1^ Movement Disorders Unit Neurology Department Vall d'Hebron University Hospital Neurodegenerative Diseases Research Group‐Vall d'Hebron Research Institute Autonomous University of Barcelona Barcelona Spain; ^2^ Pharmacy Department Vall d'Hebron University Hospital Barcelona Spain; ^3^ Magnetic Resonance Unit Department of Radiology (IDI) Vall d'Hebron University Hospital Vall d'Hebron Research Institute Autonomous University of Barcelona Barcelona Spain; ^4^ Liver Unit Vall d'Hebron University Hospital CIBERehd Vall d'Hebron Research Institute Autonomous University of Barcelona Barcelona Spain

**Keywords:** ammonium tetrathiomolybdate, efficacy, neurological symptoms, safety, Wilson's disease

## Abstract

**Objectives:**

To present our experience with ammonium tetrathiomolybdate (ATTM) in the decoppering phase treatment of Wilson's disease (WD) with neurological symptoms.

**Methods:**

An uncontrolled longitudinal study was carried out to describe a case series of five patients diagnosed of WD with neurological symptoms in our hospital over the last 5 years and receiving ATTM for 8 (or 16) weeks. Unified Wilson's Disease Rating Scale (UWDRS), Global Assessment Scale (GAS) for WD and the Brewer‐adapted Unified Huntington's Disease Rating Scale (UHDRS) for WD, magnetic resonance imaging, and monitoring for potential adverse effects were carried out in all patients before starting ATTM and 3 months later when ATTM was stopped and zinc treatment was initiated.

**Results:**

All five patients presented neurological clinical improvement in UWDRS, GAS, and Brewer‐adapted UHDRS for WD. Neuroimaging improvement was present in 2/5 patients with brain edema reduction. Mild anemia, leukopenia, and elevation of transaminases were detected in 1 patient, with complete remission after stopping ATTM for 1 week and then restarting at a half dose.

**Conclusion:**

ATTM could be a good treatment for the initial treatment of WD with neurological symptoms due to its high efficacy, with a lower rate of neurological deterioration than the drugs currently available, despite the potential adverse effects.

## Graphical abstract

Effectiveness and safety of ammonium tetrathiomolybdate in decoppering therapy for Wilson's disease with neurological symptoms, a pilot study in routine care.

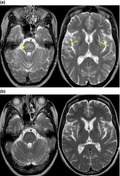

## 1 INTRODUCTION

Wilson's disease (WD) is an inherited autosomal recessive disorder caused by mutations in the ATP7B gene that encodes for a P‐type ATPase protein involved in copper transport and excretion. Recent genetic studies reported a prevalence of 1:7,026 for two mutant pathogenic ATP7B alleles (Kieffer & Medici, 2017). While the pathogenesis of WD is related to the altered function of the ATP7B copper transporter, and its accumulation, it is less clear how ATP7B mutations influence the phenotype (Medici & LaSalle, 2019).

The clinical symptoms are a result of organ dysfunction due to the direct or indirect effects of copper accumulation (Litwin et al., 2018). The severity of the symptoms varies widely and can appear anytime between early childhood and old age (Kieffer & Medici, 2017). Subsequent copper accumulation in organs, mainly liver and brain but also other organs, produces clinical manifestations that include hepatic, neurological, psychiatric, and ophthalmologic disorders (Ala, Walker, Ashkan, Dooley, & Schilsky, 2007; Kieffer & Medici, 2017; Pfeiffer, 2007).

Liver manifestations are present in 60% of patients at diagnosis and have been reported to first appear in patients aged between 9 months and over 70 years, ranging from mild elevation of liver enzymes to acute liver failure and cirrhosis (Kieffer & Medici, 2017; Litwin et al., 2018). After hepatic manifestations, neurological disorders, such as tremor, dystonia, parkinsonism, dysarthria, dysphagia, chorea, or gait disturbance (Ala et al., 2007; Kieffer & Medici, 2017; Lorincz, 2010), are the most frequent clinical symptoms of WD and may be its initial presentation in 18%–68% of patients (Czlonkowska et al., 2018). In addition to these typical and more frequent neurological symptoms of WD, other neurological symptoms may occur in the course of WD that include myoclonus, tics, headache, olfactory and taste dysfunction, neuropathies, epilepsy, restless legs syndrome, and sleep disorders (Dusek, Ltwin, & Czlonkowska, 2019). The psychiatric symptoms include anxiety, depression, disinhibition, and personality changes. The most common ophthalmological sign as a result of copper accumulation is the Kayser–Fleischer corneal ring, whereas sunflower cataracts are observed rarely, and retinal degeneration may serve as a marker of neurodegeneration (Dzieżyc‐Jaworska, Litwin, & Członkowska, 2019; Kieffer & Medici, 2017).

Patients with WD may also present with renal disturbances (including tubular dysfunction and renal calculi), cardiac involvement (a recent cardiac study has shown a higher risk of atrial fibrillation and heart failure in WD patients than in non‐WD patients), and osteoarticular involvement (including osteopenia, osteoporosis, and arthropathy, which may lead to bone fractures and joint problems mainly affecting knees and wrists; Daneshjoo & Garshasbi, 2018; Dzieżyc‐Jaworska et al., 2019).

Other manifestations of WD include autonomic system dysfunction (but involvement is subclinical in most cases) and endocrine system disturbances (which can lead to recurrent abortions, infertility, growth disruption, and parathyroid failure). However, it is possible to become pregnant for females with mild WD symptoms and for those who are compliant with therapy), hematologic disturbances (which may include acute hemolytic anemia, leucopenia, anemia, and low platelet count), and skin affectation (including lipomas and characteristic of WD skin changes like hyperpigmentation of the legs, xerosis or azure lunulae of the nails; Dzieżyc‐Jaworska et al., 2019).

For the evaluation of the neurological symptoms of WD, it has been proposed the use of few scales: the Unified Wilson's Disease Rating Scale (UWDRS; Czlonkowska et al., 2007) which evaluates the clinical motor disease, the Global Assessment Scale (GAS) for WD (Aggarwal, Aggarwal, Nagral, Jankharia, & Bhatt, 2009) which includes psychiatric symptoms and cognitive, and the Brewer‐adapted UHDRS for WD (Brewer et al., 1991, 1994) which evaluates the neurological symptoms.

Firstly described as a lethal neurological and hepatic familial disease, WD now has different drug therapies (copper chelators and zinc salts). Current guidelines (EASL, 2012; Roberts & Schilsky, 2008) recommend the use of chelators as the initial treatment of symptomatic WD patients, with the suggestion that trientine is better tolerated. However, a particular adverse event that can occur with each type of treatment is paradoxical neurological deterioration, more frequently with d‐penicillamine and trientine than zinc (Litwin et al., 2015). Worsening of neurological symptoms soon after starting treatment is reported in around 10% of patients, though much higher prevalence has also been described (Aggarwal & Bhatt, 2018) and it can be irreversible and extremely serious adverse effect that can even lead to death (Svetel, Sternic, Pejovic, & Kostic, 2001). Paradoxical neurological deterioration has prompted the discussion whether d‐penicillamine or trientine should be used in neurological WD patients and led to the search for safer treatments such as ATTM (Brewer & Askari, 2005; Brewer, Terry, Aisen, & Hill, 1987) or a more recent new formulation of TTM (Bis‐choline TTM) called WTX101 that has successfully run through a phase 2 trial (Weiss et al., 2017) and started a phase 3 trial comparing WTX101 with standard of care (chelation or zinc therapy or a combination of both chelation and zinc therapy; Brewer & Askari, 2005; Swenson, 2019).

Recent data support that patients treated with ATTM could have less probability of neurological deterioration than those treated with trientine (Appenzeller‐Herzog et al., 2019), and the most frequent side effects found were reversible increases of anemia, leukopenia, and transaminases (Brewer et al., 2006).

Our objective was to present our experience with ATTM in the treatment of WD with neurological symptoms after eight weeks of treatment by using UWDRS, GAS, and Brewer‐adapted UHDRS for WD scales.

## 2 METHODS

### 2.1 Study design and patient selection

An uncontrolled longitudinal study was carried out to describe a case series of all patients (five) who were diagnosed of WD with neurological symptoms in our hospital during the last 5 years and who were treated in the Department of Neurology with ATTM for 8 (or 16) weeks. All patients were over 18 years of age. The study was conducted in compliance with the ethical standards and was approved by the Ethics Committee for Clinical Research of Hospital Universitari Vall d'Hebron (Barcelona, Spain). Treatment with ATTM was administered as compassionate use, and all patients signed a specific informed consent to start this treatment and a specific consent to use the study data for scientific purposes.

### 2.2 Procedure and assessments

Treatment with ATTM was administered as initial decoppering treatment at a dose of 120 mg per day (20 mg three times daily with meals and 20 mg three times daily between meals) for 8 weeks. After completion of the 8‐week initial decoppering treatment with ATTM, the patients were changed to a maintenance treatment with zinc. The dose and regimen of ATTM used in our case series was described by Brewer in his initial work (Brewer et al., 1991), when he treated 6 patients with ATTM as initial decoppering treatment for 8 weeks and zinc as maintenance treatment afterward. As ATTM and zinc were not administered concomitantly, hospitalization was not needed in our case series.

All patients were followed weekly for clinical and analytical efficacy, with an anamnesis, a physical examination, measuring the levels of urinary copper in 24 hr and the levels of plasmatic copper, and monitoring for the presence of potential adverse effects: liver function alteration, anemia, or leukopenia. To ensure compliance with ATTM treatment, there was a close and exhaustive monitoring by phone calls and ambulatory visits.

A brain magnetic resonance imaging (MRI) and video recording were performed 2 weeks before starting ATTM and 3 months later when ATTM was stopped (which was 1 month after stopping ATTM) and zinc treatment was initiated.

Three scales were used: The GAS for WD, the UWDRS, and a short adaptation of the Unified Huntington's Disease Rating Scale (UHDRS; Young et al., 1986) used by Brewer in WD in several clinical trials (Brewer et al., 1991, 1994, 2003, 2006, 2009). The scales were carried out before starting ATTM and 1, 2, and 3 months later when ATTM was stopped and zinc treatment was initiated.

The GAS for WD contains two parts, GAS I to evaluate global disability in four areas: liver, cognition and behavior, motor, and osteoarticular, each item scoring severity from 0 to 5 (scores not to be summed up), and GAS II to evaluate neurological and psychiatric dysfunction with 14 items, each item scoring severity from 0 to 4 (scores to be summed up to a maximum of 56 points).

The UWDRS consists of three subscales representing three main features of clinical manifestations in WD with 35 items in total: UWDRS I to evaluate the level of consciousness, UWDRS II to evaluate the impact of the neurological symptoms on the daily life activities, and UWDRS III to evaluate the neurological signs found in the neurological examination. Each item scores severity from 0 (no symptoms) to 4 (severe affectation), except the first item (level of consciousness) where the maximum score is 3. Subsequent clinical evolution regarding neurological sequelae was extracted taking the highest score from UWDRS III: mild for scores 0 and 1, moderate for score 2, and severe for scores 3 and 4.

The Brewer scale was a short adaptation of the UHDRS to evaluate the neurological symptoms of WD.

### 2.3 Statistical analysis

The statistical analysis was descriptive and was carried out using the SPSS statistical package v17.0 for Windows.

The values from each evaluation scale were presented descriptively for each patient, before and 3 months after starting ATTM treatment.

## 3 RESULTS

We present five patients affected of WD with neurological impairment treated as compassionate use with ATTM at a dose of 120 mg per day for 8 weeks. The characteristics of the five patients are summarized in Table 1 and detailed here below.

**Table 1:** Participant characteristics

Patient	Gender	Age	Age of clinical onset	Clinical presentation	Reason for starting ATTM treatment
1	Male	21	12	Liver disease	Progression of symptoms one month after restarting treatment with d‐penicillamine
2	Female	39	15	Liver disease	Intolerance to d‐penicillamine (lupus) Progression although receiving treatment with trientine
3	Female	19	18	Neurological syndrome	WD with neurological involvement from start
4	Female	24	10	Neurological syndrome	Progression of symptoms although receiving treatment with zinc
5	Male	34	34	Neurological/psychiatric	WD with neurological involvement from start

Abbreviations: ATTM, ammonium tetrathiomolybdate; WD, Wilson's disease.

Patient 1: Man of 21 years. At the age of 12 years following an episode of acute hepatitis, he was diagnosed of WD with exclusive liver involvement and received treatment with d‐penicillamine showing good progress and remaining asymptomatic. At 18 years old, he decided, alone, to quit medication and two years later he began to have depressive symptoms and paranoid ideation. One year later, there were motor and vocal tics, with dysarthria, generalized bradykinesia, and mild dystonic posture in left upper extremity. It was then restarted treatment with d‐penicillamine with initial clinical improvement but after one month presented rapidly progressive neurological impairment. Treatment with zinc was initiated, but progressive impairment continued, and it was then decided to initiate treatment with ATTM.

Patient 2: A 39‐year‐old woman, diagnosed at 15 years of age of WD because of clinical chronic liver disease. She was treated with d‐penicillamine between the ages of 15 and 21 years, when she developed a lupus‐like syndrome due to this drug which was replaced by trientine. She remained asymptomatic until the age of 37 when she began to have a slow progressive neurological impairment with dysarthria, bradykinesia, and depressive mood. Treatment was then changed to zinc salts, but the progression of neurological impairment continued. Treatment with trientine was started again, but neurological impairment continued to progress. It was then decided to initiate therapy with ATTM. There was a neurological improvement, progressive deterioration was stopped, and the patient was stabilized. It was decided to extend treatment with ATTM eight weeks more.

Patient 3: A 19‐year‐old woman who attended our unit with dysarthria, dystonic postures in the left distal extremities (fingers and wrist bending and twisting of the foot when walking), and bilateral parkinsonism, symptoms consistent with neurological debut of WD. No previous treatment was administered. It was decided to start treatment with ATTM.

Patient 4: A 24‐year‐old woman with neurological debut at the age of 10, with progressive dysarthria, dystonia, and parkinsonism. She started treatment with d‐penicillamine, but after 1–2 months, there was a severe neurological deterioration. Treatment was changed to zinc salts, with mild improvement with sequelae, but at the age of 24, there was a new severe neurological deterioration. It was then decided to start treatment with ATTM.

Patient 5: A 34‐year‐old man, who showed an alteration in hepatic function in an ambulatory analytical control without any other manifestation or symptom at the age of 32. Diagnosed at the age of 34, when she presented neurological symptoms, dysarthria, gait disturbance, progressive parkinsonism, with lesions in MRI. Knowing the neurological deterioration risk when starting chelation with d‐penicillamine or trientine, and knowing the slow effect of zinc, it was decided to start treatment with ATTM.

All five patients received zinc salts after the 8 weeks of treatment with ATTM (16 weeks for Patient 2).

All five patients presented neurological clinical improvement between before and 3 months after starting treatment with ATTM, measured with UWDRS, GAS, and adapted Brewer scales for WD, as shown in Table 2.

**Table 2:** Results by patient

Patient	GAS I for WD pre‐ATTM	GAS I for WD post‐ATTM	GAS II for WD pre‐ATTM	GAS II for WD post‐ATTM	UWDRSI pre‐ATTM	UWDRSI post‐ATTM	UWDRSII pre‐ATTM	UWDRSII post‐ATTM	UWDRSIII pre‐ATTM	UWDRSIII post‐ATTM	Brewer scale pre‐ATTM	Brewer scale post‐ATTM	Adverse effects	Subsequent clinical evolution
1	8	4	30	12	1	0	23	8	61	20	13	9	—	Death unrelated to treatment
2	6	4	22	12	0	0	14	6	39	14	12	8	—	Moderate neurological sequelae
3	4	2	17	10	0	0	3	1	25	10	11	8	Mild transaminase elevation, anemia, and leukopenia	Mild neurological sequelae
4	4	2	20	15	0	0	4	3	15	13	7	4	—	Mild neurological sequelae
5	5	3	20	11	0	0	7	3	32	24	11	6	—	Mild neurological sequelae

Note

Postmeasurements were carried out 2–3 months after starting ATTM.

Abbreviations: ATTM, ammonium tetrathiomolybdate; GAS, Global Assessment Scale; UWDRS, Unified Wilson's Disease Rating Scale; WD, Wilson's disease.

**Figure 1:**
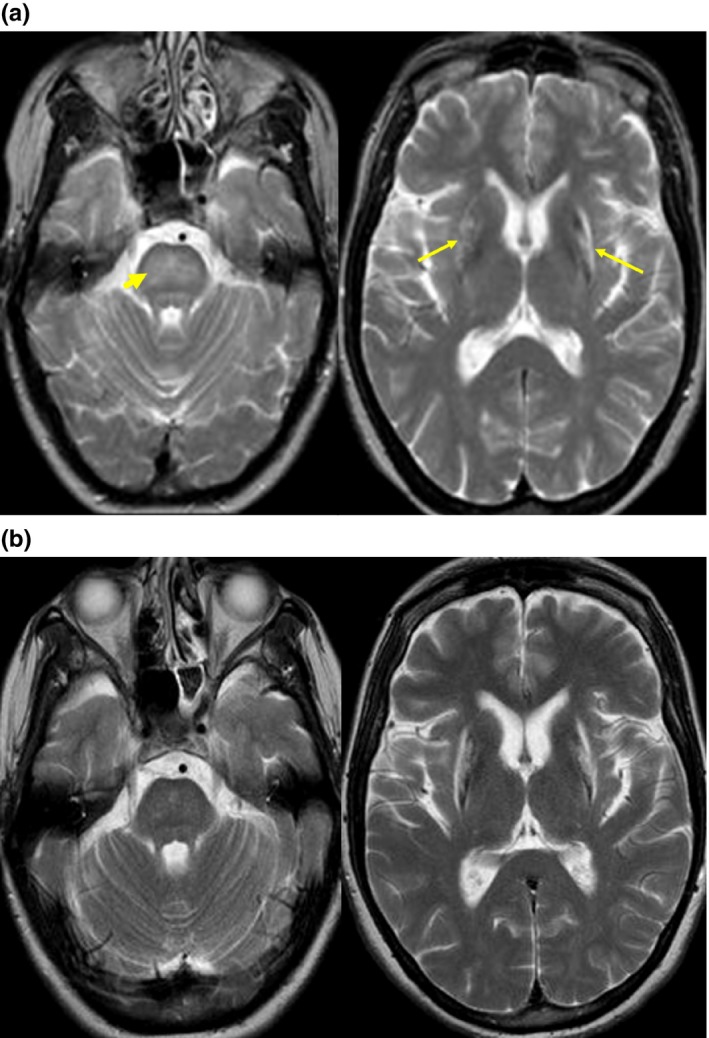
Neuroimaging improvement with brain edema reduction 3 months after starting treatment with ATTM and persistence of chronic necrotic lesions of basal ganglia. Patient 2. (a). Pre‐ATTM brain MRI. Increase in signal intensity on T2‐weighted images involving diffusely the pons (arrowhead), and the external border of lenticular nuclei (arrows). (b). Post‐ATTM brain MRI. Observe the almost complete resolution of the diffuse pontine signal abnormalities, but persistent T2 signal abnormalities associated with atrophy, likely related to necrosis, of both putamina

There was also a neuroimaging improvement in 2/5 patients (Patients 2 and 3) with brain edema reduction 3 months after starting treatment with ATTM, although chronic necrotic lesions of basal ganglia were persistent (Figure 1).

In 1/5 patients (Patient 3), a mild anemia and leukopenia together with transaminases elevation was found three weeks after starting ATTM treatment, being discontinued for 1 week and then restarted at a lower dose (60 mg/day; Table 2). There was a remission of the analytical abnormalities, and the 8‐week treatment could be finished with no more incidences.

In 1/5 patients (Patient 1), there was a transitory depression and paranoid ideation for a few days resolved spontaneously while maintaining the treatment.

No other adverse effects were found in any other patient.

In 3/5 patients (Patients 3, 4, and 5), the subsequent clinical evolution regarding neurological sequelae was mild (Table 2).

## 4 DISCUSSION

Wilson's disease with neurological symptoms, although being a potentially manageable alteration actually, it can be highly incapacitating if the treatment is inadequate or is started too late once the symptoms are present (Walshe, 2009).

In our study, none of the five patients evidenced a neurological clinical progression after starting ATTM treatment. Moreover, there was a significant neurological clinical improvement demonstrated by the three scales used to evaluate the neurological symptoms of WD, except for the UWDRS I subscale (evaluating the level of consciousness). All our patients presented a normal level of consciousness (baseline score of 0) except for one case with a baseline score of 1 (mild somnolence). This patient improved after ATTM treatment, being the level of consciousness of 0.

Clinical improvement is the expected result in WD patients starting an effective chelation therapy. A study carried out by Brewer (Brewer et al., 2003), evaluating the efficacy of ATTM, found that responding patients had a sustained significant improvement even one year after starting ATTM treatment.

In our case series, we reproduced the experience presented by Brewer in his initial work where 6 patients received ATTM as initial decoppering treatment with no worsening of acute neurological symptoms (Brewer et al., 1991). In our case series, there was a neurological clinical improvement in all five patients.

Furthermore, in our study, none of the patients presented neurological clinical progression, which also supports the evidence from the Brewer study (Brewer et al., 2006) that ATTM could lead a much lower rate of neurological progression associated with the start of the treatment than penicillamine (estimated in 50%; Brewer et al., 2003) and trientine (26%; Brewer et al., 2006), although there were very few patients. While all patients from the Brewer study in 2006 received zinc treatment together with trientine or ATTM for 8 weeks, being all patients hospitalized (probably due to the complexity of both medications administered concomitantly), patients from the Brewer study in 2003 received ATTM alone for 8 weeks as per his initial work from 1991 and our case series.

In the decoppering phase treatment of WD, chelators may induce further clinical deterioration in some treated patients. This is a paradoxical situation of great concern, whose treatment is a challenge. In our study, three of the five patients (Patients 1, 2, and 4) started ATTM treatment due to progression of the neurological symptoms although receiving treatment for WD with other drugs (penicillamine, trientine, and zinc, respectively). All three patients evidenced clinical improvement after starting ATTM treatment, which could be an indirect estimation of superiority of ATTM treatment in WD with neurological presentation with respect to the previous treatments as well as an option to rescue the neurological deterioration related to those treatments.

Concerning WD cranial lesions, some authors consider that improvement in radiological lesions in the cranial MRI is a good prognosis factor in response to the treatment (Prashkanth et al., 2005). In our study, the neuroimaging improvement found in 2 of the 5 patients after starting ATTM treatment was consistent with a clinical improvement.

The most frequent adverse effects reported after starting ATTM treatment in the Brewer study (Brewer et al., 2003) were mild myelosuppression in 5 of the 22 patients and transaminases increase in 3 cases. We found similar results in our 5 patients, where only one patient presented a mild myelosuppression (with mild anemia and leukopenia) and an increase of the hepatic enzymes. Hematological disorders are usually present between weeks 3 and 6 of the treatment (Brewer et al., 2003, 2006) and have been related to a possible effect of lack of copper due to excess chelating treatment (Brewer et al., 2003). Hepatic enzymes increase usually appears also after week 3 of treatment, and the cause is still unknown, but seems related to a direct effect of the drug. Both adverse effects disappear after a rest drug period (usually one week) and restarting the drug at half dose (60 mg/day; Brewer et al., 2003). The analytical disorders in our patient were normalized after discontinuing ATTM for 1 week, with no clinical progression evidenced during this period, and the treatment could be completed for 8 weeks with no further incidences.

ATTM was administered as initial decoppering treatment for 8 weeks. In one patient, treatment was extended eight weeks more, keeping the efficacy and safety, which is important as it allows to consider ATTM as a rescue treatment, achieving neurological improvement in patients intolerant to penicillamine or trientine treatment. ATTM would also be considered for use as maintenance treatment; however, this is hindered by the stability issues of this drug together with the too cumbersome treatment schedule with multiple daily dosing. However, the difficulties of ATTM treatment could possibly be solved with a new formulation of Bis‐choline TTM (Brewer & Askari, 2005; Weiss et al., 2017; Swenson, 2019).

In summary, there are very limited data in the literature on ATTM and Wilson's disease (which is dominated by the Brewer publications), and given this scarcity, the present case series may be of interest to the community. A new aspect is that in our experience, ATTM decoppering phase treatment for advanced neurological WD patients was effective without hospitalization and concomitant treatment with zinc. Although the study has only very few cases, it provides some more evidence to consider this drug as a good treatment for WD with neurological presentation and as rescue treatment for penicillamine, trientine, or zinc paradoxical neurological deterioration (Aggarwal et al., 2009; EASL, 2012; Roberts & Schilsky, 2008).

## 5 CONCLUSION

Our data provide some more evidence to consider ATTM could be a good treatment for the initial treatment of WD with neurological symptoms and as a rescue treatment for penicillamine, trientine, or zinc paradoxical neurological deterioration, despite the potential adverse effects which, in our experience, were mild and reversible.

## CONFLICT OF INTEREST

On behalf of all authors, the corresponding author states that there is no conflict of interest.

## AUTHOR CONTRIBUTIONS

Oriol De Fabregues designed the study and performed organization and execution; data acquisition; analysis and interpretation of data; manuscript drafting; and manuscript revision. Jaume Viñas, Antoni Palasí, and Ignasi Cardona performed execution and data acquisition. Manuel Quintana performed descriptive analysis. Cristina Auger performed the MRI. Víctor Vargas revised the manuscript.

## Data Availability

The data that support the findings of this study are available from the corresponding author upon reasonable request.
